# Patient and physician satisfaction with video consultations in dermatology: An exploratory cross‐sectional study

**DOI:** 10.1111/ddg.70035

**Published:** 2026-03-25

**Authors:** Felix von Krogh, Julia Zarnowski, Regina Treudler, Jan‐Christoph Simon, Anna‐Theresa Seitz

**Affiliations:** ^1^ Department of Dermatology Venereology and Allergology University Hospital Leipzig Leipzig Germany; ^2^ Institute of Allergology, Charité – Berlin University Medicine Medical Faculty of Free University of Berlin and Humboldt University of Berlin Berlin Germany

**Keywords:** assessment of skin findings, barriers, connection quality, impairment, satisfaction, teledermatology, video consultation

## Abstract

**Background and Objectives:**

Teledermatology has become an essential part of dermatological care, although challenges in its implementation remain. We conducted a real‐life survey on video consultations over a one‐year period to assess satisfaction and identify barriers.

**Material and Methods:**

A monocentric, questionnaire‐based study was conducted, using a self‐developed questionnaire and the German adaptation of the validated Client Satisfaction Questionnaire.

**Results:**

Of 250 scheduled consultations, 89.6% were completed. A total of 128 patients and 13 physicians participated in the survey. The majority of patients (94.5%) and physicians (94.7%) would recommend video consultations. Patients reported high levels of satisfaction, with 93% rating ease of use positively and 80.4% reporting a positive overall experience. Physicians reported high satisfaction, with 98% providing positive feedback regarding scheduling, 94.7% regarding ease of use, and 90.8% regarding overall experience. Feedback on connection stability and skin assessment varied, with connectivity issues correlating with poorer skin findings (p < 0.001). Feedback regarding connection stability and adequacy of skin assessment showed variability. Connectivity issues were significantly associated with poorer dermatologic assessment (p < 0.001). Physicians who were dissatisfied with the quality of skin assessment were less likely to recommend video consultations (p < 0.001) and reported a less favorable overall experience (p < 0.001).

**Conclusions:**

Video consultations can improve access to dermatologic care, patient and physician satisfaction, and efficient use of healthcare resources. Our real‐world data show high acceptance among patients and physicians while highlighting areas for optimization, particularly video quality and adequacy of dermatologic assessment.

## INTRODUCTION

Teledermatology has rapidly become an essential component of dermatologic care, driven by technological advancements and increasing demand for dermatologic services.^1^ The use of teledermatology has increased worldwide over the years, with a noticeable rise observed both in government‐sponsored and private healthcare systems, as well as in private practice.^2^, ^3^

Telecommunication technologies enable remote consultations between patients and dermatologists, effectively breaking down geographical barriers and improving the efficiency of dermatological services. The COVID‐19 pandemic marked a turning point in telemedical care. Dermatological care in Europe was severely impacted by the COVID‐19 pandemic, as reflected in a sharp decline in patient numbers. For example, emergency dermatological consultations at the Institute for Research, Hospitalization and Health Care in San Matteo, Italy, dropped by 79.9%, from 164 in 2019 to 33 in 2020.^4^ Similarly, hospitalizations at the Dermatology Department in Wroclaw, Poland, fell by 71.9%, from 626 to 176 during the same period.^5^

With respect to dermatologic care in Germany, the first year of the pandemic was associated with a sustained 14% reduction in inpatient skin cancer treatment, including a 16% decrease in nonmelanoma skin cancer cases and a 7% decrease in malignant melanoma cases.^6^ Furthermore, major allergy centers across German‐speaking countries reported an almost 50% decrease in the initiation of potentially life‐saving specific immunotherapy for patients with insect venom allergy.^7^ However, the COVID‐19 pandemic accelerated the implementation of teledermatology, as healthcare providers aimed to reduce in‐person visits while ensuring continuity of care.^8^, ^9^

Nevertheless, uptake of teledermatology services varies considerably. In Germany – as a representative European example – implementation remains limited: in 2021, only 7.6% of surveyed dermatologists reported using real‐time video consultations with patients.^10^ In contrast, 42.6% of private practices and 30% of academic institutions in the United States utilized similar video platforms.^11^ This substantial difference highlights the need for improvement. Potential barriers to the adoption of teledermatology could lie in limited acceptance among physicians and patients, difficulties in handling the technology, issues related to quality and reliability of video consultations and reimbursement clarity. To better understand these challenges, we conducted a survey from January to December 2021 to assess patient and physician satisfaction with video consultations in a real‐life setting. In this study, the term “real‐life” refers to the routine clinical use of video consultations as part of standard patient care, independent of the study design. Video consultations were introduced at our institution in the fourth quarter of 2020 and have since been integrated into routine outpatient care. Obtaining these insights is essential to optimize digital health services and address existing barriers.

## MATERIAL AND METHODS

### Study design

We investigated the satisfaction of patients and physicians, while also identifying potential barriers, over a one‐year period in 2021 using a monocentric, cross‐sectional, questionnaire‐based and data‐driven approach. Patients and physicians who participated in a live video consultation in the departments of allergology, general dermatology, andrology and laser department at the University Hospital Leipzig, Germany, from January 2021 to December 2021 were included after providing their informed consent. In a questionnaire‐based approach the patient and physician satisfaction was assessed after video consultations were successfully attended. Additionally, information on connection quality and stability, device and operating system, age, gender, employment status, and department were collected. Potential confounders such as computer skills and the need for assistance were considered. No formal effect modifiers were defined, though device type and department were explored as potential influencing factors. To reduce bias, we invited all participants and used standardized questionnaires. If patients or physicians did not complete the survey, their perspectives on satisfaction were not included in the satisfaction analysis. This manuscript was prepared in accordance with the STROBE (Strengthening the Reporting of Observational Studies in Epidemiology) recommendations.^12^

### Ethics Statement and Data protection

The study was conducted in accordance with the Declaration of Helsinki and approved by the local ethics committee (Medical Faculty of the University of Leipzig, approval number 029/21‐ek). All participants provided informed consent prior to participation.

Video consultations were conducted using the e‐health software Samedi, which is certified in Germany according to the European Data Protection Regulation. We used the Samedi platform for scheduling and conducting video consultations. Samedi GmbH, the platform provider, acts as the data processor on behalf of the healthcare institution, which functions as the data controller. According to Samedi's privacy policy, all personal and health‐related data are encrypted locally on users’ devices before transmission, ensuring that unencrypted data are not accessible to Samedi servers.

For data collection, all participating patients and physicians received a questionnaire via postal service or encrypted email from the clinic server. All survey responses were compiled in Excel spreadsheets and stored securely on clinic computers and internal servers ensuring that all data remained within the institution's secure network. No cloud‐based or external storage services were used. All procedures ensured compliance with applicable data protection laws and institutional policies to safeguard participant privacy and data security.

### Questionnaires

Participating patients and physicians received an internally designed questionnaire by email or postal service within a week of their online appointment at our clinic. The questionnaire contained questions on demographic data, additionally participants were also asked to rate the appointment scheduling, user‐friendliness, connection stability and overall experience using the German school grading system (1, *very good* to 6, *insufficient*). Other questions addressed the recommendation of video consultation services to friends or family (yes/no), the device used and the operating software. These questions were adapted from a previously published study that investigated satisfaction with video consultations in an ophthalmology department.^13^

In addition, patients were asked to complete the validated German version (“*Fragebogen zur Messung der Patientenzufriedenheit*”*, ZUF‐8*) of the Client Satisfaction Questionnaire (CSQ), which was originally developed by Larsen et al.^14^, ^15^ This questionnaire aims to measure patient satisfaction by assessing quality and satisfaction of treatment, fulfillment of patient needs and handling of problems. The questionnaire consists of eight items, each rated from 1 to 4. The values of all eight items are summed up and thus can assume a value of eight to 32. High values indicate a higher level of treatment satisfaction, whereas low values indicate lower level of satisfaction.

The physician survey instrument used in this study was developed in‐house to specifically assess user experience during teledermatology consultations. The questionnaire utilized the familiar German school grading system (grades from 1, *very good* to 6, *insufficient*) to facilitate intuitive responses from participating physicians. While the instrument was not formally validated, it was designed based on practical considerations relevant to the teledermatology context. A set of five related but topically diverse items, that were all rated by physicians using the same rating scale (*scale*: German school grades; *items*: scheduling of appointments, ease of use, connection stability, overall experience, assessability of skin findings) was used to assess internal consistency of the physicians’ survey using Cronbach's alpha. The analysis produced a reliability coefficient of α  =  0.806, indicating good reliability.

### Statistical analysis

Statistical analysis was performed using IBM SPSS Statistics, version 24.0. This study employed an exploratory statistical approach, as it aimed to generate initial insights into patient and clinician satisfaction with video consultations rather than test a specific hypothesis. Given the observational nature of the study and the lack of prior data for effect size estimation, no formal sample size calculation or a priori power analysis was conducted. The sample size was determined based on the number of eligible and consenting patients during the study period. Descriptive results were expressed as numbers and percentages, using means and standard deviations (SD) for continuous variable, or medians and interquartile ranges for ordinal variable. Normality was assessed using the Shapiro–Wilk test. Since the client satisfaction score (CSQ) was not normally distributed, non‐parametric tests were used. Association between continuous variables (e.g., age) were assessed using Spearman's rank correlation, differences in satisfaction scores across categorical variables with binary groups (e.g., gender) were tested using the Mann‐Whitney U test, while variables with more than two groups (e.g., employment status, operating system, device, department) were analyzed using the Kruskal‐Wallis test. A p‐value of < 0.05 was considered statistically significant.

## RESULTS

### Basic data on video consultation

From January 2021 to December 2021 a total of 250 video consultations were scheduled. Most appointments were scheduled in February (n  =  39; 15.6%), while the fewest were scheduled in October (n  =  10; 4%). In all other months, between 17 and 24 appointments (6.8–9.6%) were scheduled. Most patients had a video appointment for an allergology consultation (n  =  135; 54%), followed by general dermatology (n  =  63; 25.2%) and laser department (n  =  44; 17.6%). The fewest appointments were scheduled in the department of andrology (n  =  8; 3.2%).

Of the 250 scheduled video consultations, 224 (89.6%) were completed by both patients and physicians, corresponding to a patient no‐show rate of 10.4%. Out of 224 conducted video consultations, 206 (92%) were conducted with full audio and video quality, while an impairment of sound or image was reported in 8% of all consultations. Among all video consultations, inpatient admission was discussed in 13.2% (n  =  33), drug therapy was initiated or adjusted in 33.6% (n  =  84), and a prescription was issued in 13.6% (n  =  34).

Frequent diagnoses recorded in the context of video consultations are listed in Table 1. In the allergology department suspected food allergy (n  =  22; 8.8%), hymenoptera venom allergy (n  =  16; 6.4%) and chronic urticaria (n  =  16; 6.4%) were the most common diagnoses, whereas psoriasis (n  =  9; 3.6%), melanocytic naevus (n  =  8; 3.2%) and rosacea (n  =  7; 2.8%) were most frequent in the general outpatient clinic. Spider nevi (n  =  5; 2.0%) and hemangioma (n  =  4; 1.6%) were the most common diagnoses in laser medicine.

**TABLE 1:** Frequent diagnoses in the context of video consultations (out of 250 scheduled appointments).

Diagnosis	ICD‐10	n	%
***Allergology department***			
Suspected food allergy	T78.1	22	8.8
Hymenoptera venom allergy	T63.4	16	6.4
Chronic urticaria	L50.9	16	6.4
Suspected allergy to analgesics	Z88.6	15	6.0
Allergic rhinitis	J30.1	13	5.2
Suspected allergy to vaccination	T88.1	12	4.8
Atopic eczema	L20.8	7	2.8
Suspected allergy to antibiotics	Z88.1	7	2.8
Type IV hypersensitivity reaction to drugs	T88.7	6	2.4
Suspected allergy to local anesthesia	Z88.4	4	1.6
Allergic contact dermatitis to cosmetics	L23.2	3	1.2
***General Dermatology department***			
Psoriasis vulgaris	L40.0	9	3.6
Melanocytic naevus	D22.5	8	3.2
Rosacea	L71.8	7	2.8
Tinea unguium	B35.1	4	1.6
Lichen planopilaris	L43.8	3	1.2
Morphea	L94.0	3	1.2
***Laser department***			
Spider naevus	I78.1	5	2.0
Hemangioma	D18.0	4	1.6
Xanthelasma	H02.6	3	1.2
Hypertrichosis	L68.9	3	1.2
Café‐au‐lait spots	L81.3	3	1.2
Seborrheic keratosis	L82	3	1.2

### Demographical data of patients and physicians

All patients (n  =  224) were invited to participate in the questionnaire‐based study by email or post, with 57.1% (128/224) responding. Most of the patients that attended the video consultation and responded to our study request belonged to the allergology department (patients: 80/124; response rate 64.5%), followed by the laser department (patients: 24/39; 61.5%) and the general dermatology department (24/61; 39.3%). No patient or physician from the andrology department responded to the study request.

All 13 physicians who regularly conducted video consultations were asked to complete a questionnaire after each consultation. Treating physicians completed the questionnaires in 67.9% (152/224) of successfully conducted video consultations. Both patients and physicians were predominantly female (71.9% and 69.2%, respectively). The mean age of patients was 43.2 ± 14.0 years, while participating physicians were 31.1 ± 3.9 years. Most patients (88.2%) and physicians (96.1%) rated their self‐reported computer skills as *good* to *very good*, and the majority in both groups (patients: 93.8%, physicians: 98.0%) did not require any technical support for participating in the video consultation.

Most patients used laptops or cell phones for video consultations, while stationary computers were used least frequently. All physicians used Windows‐based stationary computers (Windows Version 10) and clinic‐based servers.

Demographic data from patients and physicians are presented in Table 2.

**TABLE 2:** Demographic characteristics of patients and physicians.

	Patients, n (%)	Physicians, n (%)
All	128 (100%)	Evaluation of 152 video consultations (100%) by 13 physicians (100%)
Male	36 (28.1%)	4 (30.8%)
Female	92 (71.9%)	9 (69.2%)
Age (years)	43.2 ± 14.0	31.1 ± 3.9

*Assistance needed*		
yes	8 (6.2%)	3 (2.0%)
no	120 (93.8%)	149/152 (98.0%)

*Computer skills (126/128)*		
Very good	53 (41.4%)	127/152 (83.6%)
Good	60 (46.9%)	19/152 (12.5%)
moderate	13 (10.2%)	6/152 (3.9%)
Poor	2 (1.6%)	0%

*Employment status*		
Employed	63 (49.2%)	Resident: 8/13 Fellow: 3/13 Senior physician: 2/13
Student/Trainee	18 (14.1%)	
Self‐employed	17 (13.3%)	
Official (civil servant)	11 (8.6%)	
Retired	7 (5.5%)	
Unemployed	4 (3.1%)	

*Department*		
Allergology	80 (62.5%)	81 (53.3%)
Laser	24 (18.8%)	36 (23.3%)
General	24 (18.8%)	35 (23.0%)

*Device*		
Laptop	66 (51.6%)	0
Cell phone	29 (22.7%)	0
Tablet	17 (13.3%)	0
Stationary Computer	16 (12.4%)	152 (100%)
***Operating system***		
Windows	82 (64.1%)	152 (100%)
Android	27 (21.1%)	0
iOS	19 (14.8%)	0

### Patient satisfaction

The scheduling of appointments received predominantly positive feedback, with 76 respondents (59.4%) rating it as *very good* and 36 (28.1%) as *good*, making up a combined 87.5% of favorable responses. The ease of use was highly rated, with 87 respondents (68%) marking it as *very good* and 32 (25%) as *good*, leading to a strong 93% positive response rate. The stability of the connection received generally positive feedback, with 73 respondents (57%) rating it as *very good* and 32 (25%) as *good*, resulting in 82% of favorable responses. The overall experience was rated *very good* by 68 respondents (53.1%) and *good* by 35 (27.3%), resulting in 80.4% positive feedback. Altogether, patients consistently rated all experience dimensions (scheduling, ease of use, connection stability, and overall experience) with a median of 1 [IQR 1–2], indicating a pronounced ceiling effect with clustering of responses at the highest satisfaction level. The majority of patients (94.5%) were willing to recommend video consultation for future use. An overview of all results can be found in Table 3.

**TABLE 3:** Patient satisfaction results.

Grade (in reference to the German school grading system)	Scheduling of appointments	Ease of use	Connection stability	Overall Experience
**All departments, n = 128**				
1 (very good)	76 (59.4%)	87 (68%)	73 (57%)	68 (53.1%)
2 (good)	36 (28.1%)	32 (25%)	32 (25%)	35 (27.3%)
3 (satisfactory)	9 (7%)	8 (6.3%)	10 (7.8%)	17 (13.3%)
4 (sufficient)	6 (4.7%)	0%	8 (6.3%)	2 (1.6%)
5 (poor)	0%	1 (0.8%)	1 (0.8%)	3 (2.3%)
6 (insufficient)	1 (0.8%)	0%	2 (1.6%)	2 (1.6%)
Median [IQR]	1 [1–2]	1 [1–2]	1 [1–2]	1 [1–2]
**Individual Departments**				
Allergology	1 [1–2]	1 [1–2]	1 [1–2]	1 [1–2]
n = 80				
Laser	1 [1–2]	1 [1–2]	1 [1–2]	1.5 [1–2]
n = 24				
General	1 [1–2]	1 [1–2]	1 [1–2]	2 [1–3]
n = 24				
**Willingness to recommend video consultations in the future**				
Yes	121 (94.5%)			
No	6 (4.7%)			
Abstention	1 (0.8%)			

The CSQ questionnaire was completed by 127/128 patients and revealed high levels of satisfaction across all categories. Positive replies (*very good* and *good*) were given by 93.0% for the quality of treatment, 91.5% for receiving desired help, 93.0% for having their needs met, 92.2% for recommending the service, 89.8% for satisfaction with received help, 88.3% for the service helping to deal with problems, 89.1% for overall satisfaction, and 91.4% for willingness to use the service again. The average CSQ score across all departments was 27.3 ± 5.1, indicating a high level of satisfaction overall. Breaking this down by department, allergology had an average score of 27.5 ± 5.3, general dermatology scored 26.6 ± 5.7, and laser 27.1 ± 4.0, reflecting consistent satisfaction levels across all areas. An overview of the CSQ results can be found in Table 4.

**TABLE 4:** Client Satisfaction Questionnaire (CSQ) results.

Items	Very good	Good	Fair	Poor
Quality of treatment^*^	53 (41.4%)	66 (51.6%)	6 (4.7%)	3 (2.3%)
Received desired help	61 (47.7%)	56 (43.8%)	7 (5.5%)	4 (3.1%)
Met your needs^*^	76 (59.4%)	43 (33.6%)	4 (3.1%)	4 (3.1%)
Recommend to friends	69 (53.9%)	49 (38.3%)	5 (3.9%)	5 (3.9%)
Satisfaction with help received	64 (50%)	51 (39.8%)	6 (4.7%)	6 (4.7%)
Helped to better deal with problems	70 (54.7%)	43 (33.6%)	8 (6.3%)	6 (4.7%)
Overall satisfaction^*^	66 (51.6%)	48 (37.5%)	7 (5.5%)	6 (4.7%)
Willingness to use the service again^*^	67 (52.3%)	50 (39.1%)	7 (5.5%)	3 (2.3%)

Average CSQ Score (all departments)	27.3 ± 5.1 (95% CI: 26.4–28.2)			
Average CSQ Score (allergology)	27.5 ± 5.3 (95% CI: 26.3–28.7)			
Average CSQ Score (general dermatology)	26.6 ± 5.7 (95% CI: 24.3–28.9)			
Average CSQ Score (laser)	27.1 ± 4.0 (95% CI: 25.5–28.7)			

*Abbr*.: CSQ: Client Satisfaction Questionnaire (German Version)

* 127/128 participants

In further analyses, we wanted to investigate possible correlations between overall patient satisfaction and factors such as age, gender, employment status, operating system, device used or department. Correlation analysis revealed no significant association between CSQ and age (p  =  0.74, r_s_  =  0.03), Mann‐Whitney U test showed no significant difference in satisfaction scores between the gender groups (p  =  0.59, U  =  1718, Z  =  0.53). There was no statistically significant difference in CSQ scores related to employment status (p  =  0.27, χ^2^(7)  =  8.81), device used (p  =  0.30, χ^2^(3)  =  3.69), operating system (p  =  0.57, χ^2^(1)  =  0.32), department (p  =  0.52, χ^2^(2)  =  1.32) or self‐reported computer skills (p  =  0.80, χ^2^(3)  =  1.00). Age was significantly associated with the perceived level of computer skills (p  =  0.002, χ^2^(3)  =  14.68), although the need for assistance was not associated with age in our patient cohort (p  =  0.60, U  =  532.5, Z  =  0.52). Overall satisfaction showed a positive association with a stable audio and video connection (p  =  0.018). Interestingly, although no significant association was found between overall satisfaction and the device chosen, the perceived ease of use was significantly associated with the device (p  =  0.011), being highest in cell phone users and lowest in stationary computer users.

### Physician satisfaction

The physician satisfaction survey revealed strong positive results, especially in appointment scheduling, ease of use and overall experience, while connection stability and assessability of skin findings received more varied feedback. For scheduling of appointments, 86.2% of respondents rated it as *very good* and 11.8% as *good*, resulting in a positive response rate of 98%. The ease of use achieved a positive response rate of 94.7%, consisting of 86.8% *very good* ratings and 7.9% *good* ratings. For connection stability, 66.4% rated it as *very good*, 19.7% *good*, 9.9% *satisfactory*, and 2.6% *sufficient*. A small number rated it *poor* or *insufficient* (0.7%, respectively). For the overall experience, 72.4% of physicians rated it as *very good* and 18.4% as *good*, resulting in a 90.8% positive response rate. The feedback for assessability of skin findings was more varied, although this was not equally relevant across all departments as physicians were requested to fill out their experience in skin assessability only when applicable to their consultation. Consequently, the response rate to this subitem was 9.9% in allergology (n  =  8/81), 45.7% in general dermatology (n  =  16/35) and 100% in the laser department (100%, n  =  36/36). Altogether, 13.3% rated it as *very good*, 13.3% as *good*, 20% as *satisfactory*, 21.7% as *sufficient*, 25% as *poor*, and 6.7% as *insufficient*. The overall grade was the worst achieved of all items, with a median of 4 [IQR 2–5]. The majority of physicians (94.7%) were willing to recommend the video consultation for future use. An overview of these findings can be found in Table 5.

**TABLE 5:** Physician satisfaction results.

	Scheduling of appointments	Ease of use	Connection stability	Overall Experience	Assessability of skin findings
***Ove*rall grades across departments (Median, IQR and p*ercentage distribution of individual grades)***					
Grade (in reference to the German school grading system)	1 [1–1]	1 [1–1]	1 [1–2]	1 [1–2]	4 [2–5]
1 (very good)	131 (86.2%)	132 (86.8%)	101 (66.4%)	110 (72.4%)	8 (13.3%)
2 (good)	18 (11.8%)	12 (7.9%)	30 (19.7%)	23 (18.4%)	8 (13.3%)
3 (satisfactory)	1 (0.7%)	5 (3.3%)	15 (9.9%)	7 (4.6%)	12 (20%)
4 (sufficient)	1 (0.7%)	3 (2.0%)	4 (2.6%)	5 (3.3%)	13 (21.7%)
5 (poor)	1 (0.7%)	0%	1 (0.7%)	2 (1.3%)	15 (25%)
6 (insufficient)	0%	0%	1 (0.7%)	0%	4 (6.7%)
	n = 152				n = 60
**Grades over individual departments (Median, IQR)**					
Allergology	1 [1–1]	1 [1–1]	1 [1–1]	1 [1–1]	3.5 [2–4.75]
	n = 81				n = 8
Laser	1 [1–1.75]	1 [1–1]	2 [1–3]	1 [1–3]	4 [3–5]
	n = 36				
General	1 [1–1]	1 [1–1]	1 [1–1]	1 [1–1]	3 [1–5]
	n = 35				n = 16
**Willingness to recommend video consultations in the future**					
Yes	144 (94.7%)				
No	8 (5.3%)				
Abstention	0 (0.0%)				

Although the overall physician experience had a median score of 1, indicating a *very good* overall satisfaction, the interquartile range of the laser department [1–3] reveals a lower satisfaction. This was primarily driven due to the need for reliable diagnostic assessment, as therapeutic decisions rely heavily on the evaluability of skin lesions.

When considering physician experience, overall experience and recommendation correlated strongly with the department in which the video consultation was performed (p < 0.001). Video consultations with patients seeking laser therapy showed significantly poorer outcomes regarding connectivity (p < 0.001), overall experience (p < 0.001), and likelihood of recommendation (p < 0.001) compared with consultations conducted by the allergology department. In addition, connectivity issues (p < 0.001) correlated with poorer ratings regarding the assessability of skin findings. Physicians who rated the skin assessment as poor were less likely to recommend the video consultation (p < 0.001) and had a poorer overall experience (p < 0.001).

### Qualitative Analysis of Commentary Sections

Both the patient and physician surveys included a free‐text comment section in which individual remarks were collected. In total, 49 comments were received from patients and 14 from physicians. Perceived benefits and experienced limitations are summarized in Table 6. Comments that were mentioned repeatedly are highlighted in *italics*.

**TABLE 6:** Summary of patient and physician commentaries.

	Patients Patients: 49/128 commentaries	Physicians Physicians: 14/152 commentaries
Audio	− *Audio impairment* (poor quality, echoing), Headset for physicians recommended	− *Patient's microphone malfunctioned, no communication possible*
Video	− *Resolution issues (Skin assessment missing; unable to inquire about other skin changes*	− Video connection was unstable or unavailable when patients used mobile data instead of Wi‐Fi. − Resolution issues (poor video/image quality) leading to limited skin assessment.
Others	− Lack of continuity: different doctors conducting consultations led to reduced trust − *Preference for in‐person conversations* − Video consultations seen as equivalent to phone calls; no added benefit − *Increased flexibility for appointments (e.g., during vacation or working abroad)* − *Time saved due to reduced travel and waiting*	− No time savings were observed due to time‐consuming post‐processing tasks such as SAP entry and sending prescriptions. − Consultations were very time‐consuming when patients faced IT problems, often requiring phone assistance. − *Lack of dermatoscopy and tactile examination hindered diagnostic evaluation* − *Video consultations are unsuitable for skin assessment related to laser treatment indications, evaluating new skin changes, or monitoring progression of existing lesions*. − *Melanocytic lesions were particularly difficult to assess via video*.

Patients frequently reported audio‐related problems, whereas physicians more often described an impairment in video quality. Positive comments regarding increased flexibility and time savings were predominantly expressed by patients. In contrast, physicians more frequently emphasized drawbacks, including time‐consuming post‐consultation documentation and perceived limitations in diagnostic assessment.

Since all physicians used the same device and platform (stationary clinic computers), we analyzed the association between patients’ devices, operating systems, and reported connection stability. Neither the type of device (p  =  0.33, r_s_  =  0.088) nor the operating system (p  =  0.83, r_s_  =  0.019) showed a significant correlation with reported connection stability.

## DISCUSSION

Our study aimed to evaluate the acceptance, satisfaction, and perceived barriers among both patients and physicians with regard to video consultations, with the objective of optimizing the implementation of teledermatology services. Overall, teledermatology provides a broad range of applications and may be used for initial consultations, follow‐up visits, and the long‐term management of chronic dermatologic conditions. This solution also serves as an effective triage tool, helping physicians identify patients who require in‐person care.^16^, ^17^ The use of teledermatology has shown to improve access to dermatological services, making it easier for patients to reach specialists particularly in rural settings where specialist access is often limited.^18^, ^19^, ^20^ However, successful integration depends on high acceptance by both physicians and patients and requires a thorough analysis of potential risks and barriers.

Our study provides real‐world data and demonstrates a high level of satisfaction among both patients and physicians, as reflected by the proportion of 94.5% of patients and 94.7% of physicians who indicated that they would recommend the service. This level of acceptance is crucial for the successful adoption of teledermatology in clinical practice, enabling healthcare providers to deliver timely and effective care to a wide range of patients. A detailed analysis demonstrated particularly high levels of patient satisfaction with appointment scheduling and ease of use. Overall, 80.4% of respondents rated their experience positively. The CSQ questionnaire further confirmed high satisfaction levels across all evaluated dimensions. Additionally, a strong willingness to reuse the service was evident, with 91.4% of patients indicating they would use it again. A similarly high level of patient satisfaction was reported by Pearlman et al., with 88.9% of patients indicating overall satisfaction with their teledermatology experience.^21^ In a study by Hindelang et al., 92% of participants with chronic spontaneous urticaria regarded a digital care concept as a promising alternative to conventional consultations, highlighting its convenience and suitability for the management of chronic conditions.^22^ In a study by Ruggiero et al., high levels of satisfaction were observed among patients using both video‐ and telephone‐based teledermatology services. Patients who participated in video consultations reported higher satisfaction than those who received telephone‐based consultations alone; however, this difference did not reach statistical significance.^23^ A possible explanation for this could be that video‐based consultations enable a more comparable experience to an actual in‐person visit than in a phone consultation. Additional data from systematic reviews that address teledermatology implementation report a high patient satisfaction of ≥80%, largely driven by convenience and reduction of waiting times.^24^, ^25^, ^26^, ^27^

Physician satisfaction in our study was even higher than patient satisfaction in areas such as appointment scheduling, ease of use, connection stability, and overall experience. These findings are consistent with those of Mounessa et al., who likewise reported high levels of provider satisfaction with teledermatology^28^. However, variability was observed with respect to the assessability of cutaneous findings. Lower ratings were observed in laser therapy compared to allergology, likely due to the greater reliance on detailed visual assessment in laser treatments, whereas allergology often focuses on anamnesis and laboratory result discussions. Furthermore, our study demonstrated a clear association between connection quality, the ability to assess skin findings, and physicians' willingness to recommend video consultations. This highlights the critical importance of high‐resolution video transmission, connection stability, and reliable devices, particularly for visually dependent specialties.

From a clinical perspective, the limitations of remote dermatologic assessment must be acknowledged. The inability to perform tactile examinations or use dermoscopy can constrain diagnostic accuracy. Therefore, teledermatology may be best suited for triage and follow‐up care, whereas complex or ambiguous cases may still require in‐person consultations. Similarly, insights from systematic reviews elaborate on the importance of technological quality, as inadequate visual assessment consistently predicted lower satisfaction and diagnostic confidence.^24^, ^25^, ^27^ Our findings must also be interpreted in light of the physician profile as 60% of participants were residents. While this group may be more open to digital innovations, their relatively limited clinical experience could influence confidence in remote decision‐making.

To ensure effective teledermatology, it may be advisable to adopt the approach proposed by Pearlman et al., who suggest that a hybrid model of teledermatology could be advantageous until video resolution approximates the quality of consumer‐level digital photography.^21^ Beyond advanced imaging, the integration of technical advancements – including dermoscopy, confocal microscopy, and the incorporation of artificial intelligence (AI), such as large language models (LLMs), into telemedicine workflows – may offer further potential to enhance teledermatology.^29^, ^30^

Implementation of teledermatology into existing healthcare workflows also presents logistical challenges.^25^, ^26^, ^27^ Ongoing education and training for both patients and physicians is essential to ensure the effective use of these tools. Beyond the need for high‐quality imaging, it is crucial that patients are trained to produce suitable images and navigate teledermatology platforms effectively. Similarly, healthcare providers must develop the skills to use these systems proficiently and to interpret patient‐submitted visual material accurately. Furthermore, teledermatology should be implemented with sensitivity to local cultural contexts needs and resource constraints.^31^

Legal and ethical concerns – including data protection, liability, and platform governance – remain unresolved and require further legislative attention to ensure safe, equitable use. Economic and quality‐related concerns are equally important. There is a risk that efficiency pressures may prioritize speed over care quality, underlining the need for regulatory frameworks that safeguard both patient outcomes and provider responsibilities. Other factors impacting the use of teledermatology include the sustainability of platform setup, the digital literacy of users, consistent internet and mobile network access, patient preferences for face‐to‐face interaction, privacy and data protection concerns, and the establishment of a viable reimbursement system.^31^, ^32^ Possible benefits and drawbacks for both patients and healthcare providers that have been discussed regarding video consultations are summarized in Table 7, offering further insight into the practical implications of teledermatology in routine care.

**TABLE 7:** Advantages and disadvantages of video consultations.

Benefits for patients	Drawbacks for patients
− time and cost savings − quicker access to consultations − access to consultations during special conditions (e.g., pandemic, physical/mental limitations, business trips) − privacy and comfort (e.g., no need to leave home or workplace) − −improved access in rural areas	− initial consultations can be challenging (limited diagnostic options, establishing trust remotely) − requires adequate device, technical setup and skills − privacy/data concerns
**Benefits for healthcare providers**	**Drawbacks for healthcare providers**
− conserves resources by reducing in‐person visits − time and cost savings − enables remote work − reduced infection risk − improved access in rural areas	− initial consultations can be challenging (limited diagnostic options, establishing trust remotely) − requires adequate device, technical setup and skills − privacy/data concerns

While our study offers a valuable snapshot in time, the field of teledermatology continues to evolve. Ongoing research is essential to keep pace with advances in technology, changes in clinical workflows, and shifting user expectations. Our real‐world data demonstrate high satisfaction among both patients and physicians, underscoring the clinical relevance and utility of video consultations in dermatology. Additionally, the study highlights areas for improvement – particularly in video quality and the assessment of skin findings. As teledermatology advances, continuous evaluation and adaptation are crucial to fully realize its potential for patients and healthcare systems. Notably, evidence gaps remain, with a scarcity of randomized implementation studies, cost‐effectiveness analyses, and standardized satisfaction instruments.^26^

### Limitations

This single‐center study on video consultations in dermatology has several limitations that should be considered when interpreting the findings. The focus on selected subspecialties (e.g., laser group) and a modest sample size (128 patients, 13 physicians) limits generalizability and statistical power. Selection bias may have led to an overrepresentation of patients favorable to telemedicine. Future multi‐center studies with larger, more diverse sample sizes are needed to strengthen and validate these results.

Physician data were collected using an unvalidated scale; Although internal consistency was acceptable, as assessed by Cronbach's alpha, the absence of formal psychometric validation limits the interpretability of the findings. Additionally, the analysis did not account for clustering of responses within physicians, as multiple questionnaires per physician violated assumptions of independence. Ethical and data constraints prevented the use of mixed‐effects models or generalized estimating equations, possibly underestimating standard errors and inflating Type I error risk. Thus, p‐values should be interpreted cautiously.

Statistical analyses were exploratory and based on non‐parametric methods without corrections for multiple testing. This approach balances minimizing false negatives with an increased risk of false positives, underscoring the need for confirmatory studies.

The study lacked objective economic or clinical efficiency data, and future research should incorporate assessments of cost‐effectiveness and long‐term sustainability. Furthermore, only 57% of all patients responded to our survey request, with no formal non‐responder analysis possible due to lack of consent and data, raising the potential for response bias favoring satisfied patients. A lower response rate in general dermatology, possibly influenced by higher private payer percentages, may also affect representativeness.

## CONFLICT OF INTEREST STATEMENT

None.
